# Septic Poison

**Published:** 1888-01

**Authors:** L. D. Hill

**Affiliations:** Webberville, Texas


### SEPTIC FEVER.

*By L. D. Hill, M. D., Webberville, Texas.*

Read at the meeting of the Austin District Medical Society Dec. 8, 887, and contributed to the JOURNAL by vote of the Publishing Committee.

IN calling your attention to Septic Fever, I am well aware that I am entering a field of the widest interest, that has received the closest attention of the best minds in the medical profession for many years, and still is less understood to-day by the profession than any other subject of such importance. A correct knowledge of its origin, propagation and prevention is probably necessary for the adoption of proper hygienic measures for the protection of individuals, communities and nations.

At the beginning of this investigation we are met with a varied nomenclature and classification of diseases, acknowledged to owe their origin to the same poison. Dr. S. D. Gross, in his great work on surgery, volume 1, page 139, under the head of septicæmia and pyæmia, says:

"The intimate nature of the poison of septicæmia and pyaemia is entirely unknown. The probability is that it is similar to, if not identical, wifh the poisons of traumatic, or surgical fever, and kindred affections;" and on page 140, same volume, after summing up the evidence of all the experimenters up to date, says: "To sum up my own views of the nature of the septic poison, I may state that I believe that the infective material is sepsin, or the chemical product of the decomposition of the tissues, and that the schizomycites, which have been shown to be present in the blood and organs of septic and pyæmic animals and men, as well as the corpuscles of putrefying blood and pus, act merely as carriers and disseminators of the poison."

Dr. B. A. Watson, in Peppers' System of Practice of Medicine, volume 1, page 955, says: "Septic poisoning may be justly regarded as a single chain composed of many links. Take for example a case of amputation of the thigh, followed within a few hours by traumatic fever, later by septicæmia; afterwards there may be developed secondary fever, formation of ichorous pus with absorption and its constituents; pyæmia, accompanied by embolism, thrombosis, abscess in lungs, liver, etc. To these may also occasionally be added phlebitis, and inflammation of the joints, terminating speedily in suppuration. This chain may, in this case, be further lengthened or varied with traumatic erysipelas, or with hospital gangrene. In fact, the variations in these cases are very numerous, and all these conditions, with many others, are due to septic blood poisoning."

Dr. John S. Billings, surgeon U. S. A., in Ziemsen's Cyclopedia of the Practice of Medicine, vol. xviii, page 14–25, says: "From the parasitic blood diseases as typified by splenic fever, and the contagium diseases as typified by scarlatina, we may pass to an intermediate group in which the disease seems due to a poison produced by microphytes, but in which the production does not occur, or at least does not usually occur within the body. Of these there are two forms. The first is of great importance, in part because it occurs everywhere and at all times, constituting an ever present danger, especially in surgical and puerperal cases; in fact, because we now have good reason to believe that it can be prevented in the great majority of instances. This is septicæmia.

"The use of the terms septic poison, septic bacteria, septic infusoria, etc., is somewhat confusing as found in modern literature. Septic, by many writers, is used to mean belonging to, or connected with putrefaction, and by septic bacteria are meant those microdemes which are found in putrefying fluids. Such microdemes may or may not be capable of causing the fever and embolic phenomena characteristic of septicæmia. It cannot be too clearly understood that there are many kinds of microdemes; that a bacterium is simply one form of many microdemes, and that to assert that a disease is due to bacteria is to assert only that the disease is due to some kind of microdeme at the stage of its development when it is a rod. As rapidly as we are able to find distinguishing characteristics for classes in these minute bodies, let them be labeled with distinctive names, but let it not be supposed that the name of bacteria implies any identification or classification.

"For instance, if one says that bacteria are the sole cause of putrefaction, I must deny it, for I have seen putrefaction in the absence of bacteria, but I have never seen it in the absence of microdemes."

"Septicæmia, as defined by Dr. Burdon Sanderson, is a constitutional disorder of limited duration, produced by the entrance into the blood stream of a certain quantity of septic material.

"This septic poison may be separated from putrefying material by chemical process, and obtained in the form of a transparent fluid.

"This fluid contains no germs in the sense that a germ is that which produces an organism, but it does contain a specific virulent something which can be separated from it by a porcelain filter, and which does not multiply and reproduce itself. The sepsin is not then the cause, but the result of putrefaction, and when introduced into the living body its effects are in proportion to the quantity introduced like those of a chemical poison. In order that the microdeme may become the starting point of the septic or pyæmic process in the living organism it seems necessary that it shall have been derived from, or come in contact with, some specific source of contagion, or else (but such cases are certainly in the minority) that the condition of some part of the living body shall have been so far changed from its normal vitality that the microdeme can flourish in it as in dead organic matter."

"In either case, in addition to the growth of microphytes and the production of their specific products, it is necessary to the production of the septic phenomena that the condition of the tissues and vessels shall be such as to permit the entrance of the septic poison into the blood. Hence, two methods of prevention of blood poisoning after surgical operations have given good results; the first being the well known method of Lister, with its many modifications, which aims at totally preventing the contact of living microphytes with the injured surface; the second being the air dressing, which aims at the freest possible exposure, in order that the dead and septic matters may easily and rapidly escape.

"So long as only the ordinary microdemes of all air and water are present, the difference between the results obtained by these apparently opposite methods is not great; but so soon as the element of specific contagium comes in the only hope of safety lies in the absolute exclusion."

Dr. Sanderson has also shown that while the property of producing the septic poison is not possessed by the ordinary bacterial forms, or at least not to a marked degree, it may easily be developed in great potency by the injection of fluids containing these forms into the peritoneal cavity of the Guinea pig, re-injecting the effused fluids thus produced into a second animal, and so on, each successive production of fluid increasing in its power of producing rapid septic poisoning.

Dr. T.Gaillard Thomas defines puerperal fever as "puerperal septicæmia." It matters not, he advises, whether it assumes a form of phlebitis, cellulitis, etc., the essence of the disorder is the absorption of poison into the blood of parturient woman through some solution of continuity in the tissues of the genital tract. Dr. Paul F. Munde has always believed puerperal fever is puerperal septicæmia, (in the majority of cases) due to the absorption of poisonous matter from the genital tract. Dr. Bauer, in Ziemsen's Cyclopedia Practice of Medicine, vol. viii, page 278, says: "If we put the question in this way, whether microbific poison may be actually developed and may proliferate within the person of the individual, we must, with the facts we have before us, answer in the affirmative, and on page 277, "according to my idea, the more frequent occurrence of puerperal diseases in lying in institutions, as compared with the general experieuce in private practice, does not altogether result from the increased tendency to communicate the disease through the medium of those who examine and attend the cases."

Pyæmia in surgical stations also is not always communicated by the hands of the dresser, and washing the hands in carbolic acid does not put a stop to its spread. Dr. Playfair recently introduced into the British Medical Association the subject of the prevention of puerperal fever. In which he says "To prevent puerperal fever we must have an intelligent idea of what it is, how it originates, and how it may be conveyed to the patient. Unless we are agreed on these points we are not likely to come to any conclusion of value. I take it to be now almost universally admitted that puerperal septicæmia is practically the same thing as surgical septicæmia, a disease caused by a poison absorbed through the genital tract into the system, which poison may either originate *de novo*, or be conveyed to the patient from without by septic matter being brought in contact with her." In this discussion Dr. Shaw had practiced in London for thirteen years, and had 1000 cases of labor. He never washed out a vagina, disinfected his forceps, nor lost a case from puerperal septicæmia. Dr. John W. Byers, in the same discussion, after giving some statistics showing the great mortality of puerperal fever, said it was impossible to take measures for its prevention until we had clear ideas as to its causation. He discussed the various views, and gave his reasons for believing it was blood poisoning, analogous to septicæmia met with in surgical practice. Taking this view, he believed there were sources by which the poison might be introduced into the system of a lying in woman. First, sewer gas, as in unhealthy homes; second, nurse. third, accoucheur. He drew attention to the cases in which a decomposing blood clot or placenta might be the source of the disease, but believed that even in these the decomposing mass in the uterus only acted as a nidus in which the poison from without found a suitable soil.

In the same discussion, Mr. Lawson Tait stated that he had performed 306 laparotomies with only two deaths; several were for septicæmia.

The question is, what can we find in common among all these writers?

All agree, that the cause is to be found in putrefying animal tissues. All agree that some form of microdemes is present in the septic fluid. All agree that there is a stage in the septic process in which the septic poison is not absorbed into the blood stream with microdemes in it. All agree that there is a stage of development, at which it is absorbed, to be blood containing microdemes and that the disease is specific, and may be communicated from the infected to a healthy wound by actual contact with the infected wound or its fluids.

Some believe, that the poison is sepsin; others, that the true cause is bacteria; while others maintain that bacteria are only the carriers of the sepsin. Some believe that it may be communicated through the air, especially of foul surgical stations and lying-in institutions. Some believe, it may originate *de novo* in putrefying surgical wounds, or the genital tract of parturient women; while others believe, that it is always contagious, and originates in a *something* that comes from a like disease.

I saw much of the effect of septic poisoning in gunshot wounds during the war, in the hospitals at Warrenton and Richmond, Virginia.

After the second battle of Manassas, there were about twenty-five hundred wounded Confederate, and a like number of Federal soldiers left at Warrenton, Va. They were crowded into store houses, school houses, the court house, and every available room in the town. Hospital stores were scarce, rations were short and inappropriate; nurses inexperienced and few in number; the modern antiseptic treatment unknown. Soon the effects of septic poison was seen in these crowded hospitals—in the form of septic toxæmia, traumatic erysipelas, pyæmia and hospital gangrene, more dying from the effects of these diseases than fell upon the battle field.

My experience in the Texas hospital at Richmond, was about the same; it was the headquarters of the sick and wounded of Hood's Texas Brigade, and had in it, at various times, every form of septic disease connected with wounds. I do not know where it originated, in Warrenton or Richmond. I only know that some of the hospitals were closed in both places on account of the virulence of the poison in them. I also know that most of those who went to the hospital to shirk duty, died from diseases contracted there, mostly diarrhœa, typhoid fever, dysentery and erysipelas, with no traumatic lesion as a starting point. This is the experience of all who served in the Confederate hospitals. At this point, I wish to state, what I believe, to be a general surgical principle; that there is as much in the *operator*, as in antiseptics.

The amputations and other surgical operations performed by Dr. Darby, of the Hampton Legion. South Carolina, Dr. Robert Battey, 19th Georgia, and Dr. J. C. Jones, Fourth Texas regiment, all in our division, and whose field operations fell into my hands, for after-treatment at various times and places, were all clean operations. The flaps cut smooth and to fit, so as to secure perfect coaptation, and neatly dressed, generally healed by first intention; while other operations performed by surgeons in the same division, and who operated under like circumstances—often in the same field-hospital—were haggled, with portions of wounded and bruised tissues left partially deprived of their connection with the circulation, to suppurate in the wound, securing only partial coaptation, and carelessly dressed, were sure to have pus formed in them. These wounds generally sloughed, giving much trouble in their after-treatment.

The first healed without antiseptics, the others suppurated without them, and I think would have done so under an inch thickness of antiseptic gauze and two folds of silk oilcloth.

The chemical essentials of putrefaction are heat, air and water; the heat between 60 and 90° Fah. Freezing cold, or heat at the boiling point, checks or prevents oxidation; if all these factors are excluded, there can be no oxidation, no putrefaction, and no fermentation. I think that microdemes are too universally diffused to be the only cause of putrefaction, while it is proven, that they are always present in putrefaction and fermentation,—which process requires the presence of air and moisture for their starting point,—and microdemes are found in all air and water. The modern surgical dressing proposes by antiseptic gauze and silk oil cloth to exclude the entrance of all microdemes, but in doing so, they also exclude air and water, the great carriers of these microphytes, and two known essentials of chemical change upon which the formation of sepsin and the ptomaines may depend.

That the use of bichloride of mercury and other antiseptics prevents the formation and absorption of septic poison seems to be proven, (at least in the genital tract), but how they do it has not been demonstrated. Do they act as an antidote to a chemical poison? Do they act as germicides, or do they give such tone to the tissues as to render them capable of resisting the poison? These are questions that have never been satisfactorily answered.

Modern pathology, unlike ancient hypothesis, requires demonstration, and while it is safe and right for us to hold our individual views, as to what future investigation may prove, it is unwise and unscientific to accept as demonstrated, what has only probability to sustain it. The Italian scientist, Prof. Solme, has found in canned meats, when exposed to air, and after putrefaction, microscopic bodies which he calls ptomaines. The poison resulting from the putrefaction, due to the presence of ptomaines, is like that of the Indian poison curare.

Sepsin has been found in fermenting vegetable, and putrefying animal fluids, the poison produced by ptomaines in canned meats. Are they all chemical combinations of the same poison in different proportions? In this investigation the chemist is met with the chemical law of isomerism, where animal substances of the same chemical elements in the same proportions, are not the same in their effects upon living organisms, and nothing but long, close and well guarded experiments by competent chemists, can solve this vexed question satisfactorily, and every advance in animal chemistry will aid in this investigation.

The minute germs that fill the dust of the earth, and the air and water, that surrounds it,—according to Professor Tyndall,—need to be arranged into classes, orders, genera, species and varieties, as they probably will be in the future. We may then know what is a germ, and what the complete organized individual, through how many stages of development each passes from the germ to maturity, how to recognize them in each stage, and where to look for them in each form. We may find, that as they have been attenuated and rendered harmless, as claimed by Pasteur and others, by cultivation that they may be concentrated and rendered more virulent by cultivation also ; and that traumatic lesions, and the genital tract of parturient women, are the very best fields for increasing their powers for evil.

Much has been accomplished by antiseptics. Surgical operations have been shorn of many of their dangers and parturition of much of its fatality ; but how to treat pyæmic and septic diseases, when fully developed, has not yet been satisfactorily demonstrated.

To sum up my own views on septic poisoning ; I believe that septic poison originates in putrefying animal matter, either within or without the system ; that it may originate *de novo* in surgical wounds, in the genital tract of parturient women, in human excrement and in dissecting rooms ; and when fully developed, may be transmitted from an unhealthy to a healthy wound through the foul air of surgical stations and lyingin institutions, through water contaminated with sewer gas ; but that it is generally disseminated by the fluids of an infected wound coming in contact with healthy wounds ; transmitted from one to the other by the hands of nurses, surgeons, and accoucheurs, and by the careless use of surgical and gynæcological instruments, sponges, dressing material, etc., in hospitals and lyingin institutions. That the ordinary microdemes of all air and water, innocent in themselves, can by actual contact with putrefying masses become so impregnated with the poison upon which they subsist as to render them virulent in their nature; in fact, they become as sepsin, and not only act as carriers of the poison, but are themselves infectious and contaminating to the system.

To develop septic poison in the system, or for it to be absorbed, when generated out of it, I believe there must be a pathological state of the blood and tissues, not yet fully understood ; but known to be deficient in red blood corpuscles, and an abnormal increase in the white blood corpuscles and fibrin.

The field for investigation is wide and inviting to future investigators.

Let the chemist, the naturalist and the pathologist continue to investigate the dust of the earth, the air we breathe, the water we drink, and the food we eat, and demonstrate what they contain of chemical elements, living organisms and poisonous gases, and their several effects upon the human organism and how to place the human body on such a high plane of physiological functioning as to prevent their propagation or development; the antidote for each chemical poison, the germicide for each specific germ and how to eliminate, neutralize or prevent the formation of each poisonous gas; then we will fully comprehend septic poisoning, and not until then can we adopt intelligent prophylaxis and scientific treatment for all the forms of septic fever, from the diarrhœa of the dissecting room so common among medical students to the complete saturation of blood and tissues in pyæmia and hospital gangrene.

